# Supplementary material for: Genomic Regions Associated with the Control of Flowering Time in Durum Wheat
Source: Plants (Basel). 2020 Nov 24;9(12):1628. doi: 10.3390/plants9121628 (PMC7759329; doi:10.3390/plants9121628)
Supplement: Supplementary file 1 [file plants-09-01628-s001.pdf]

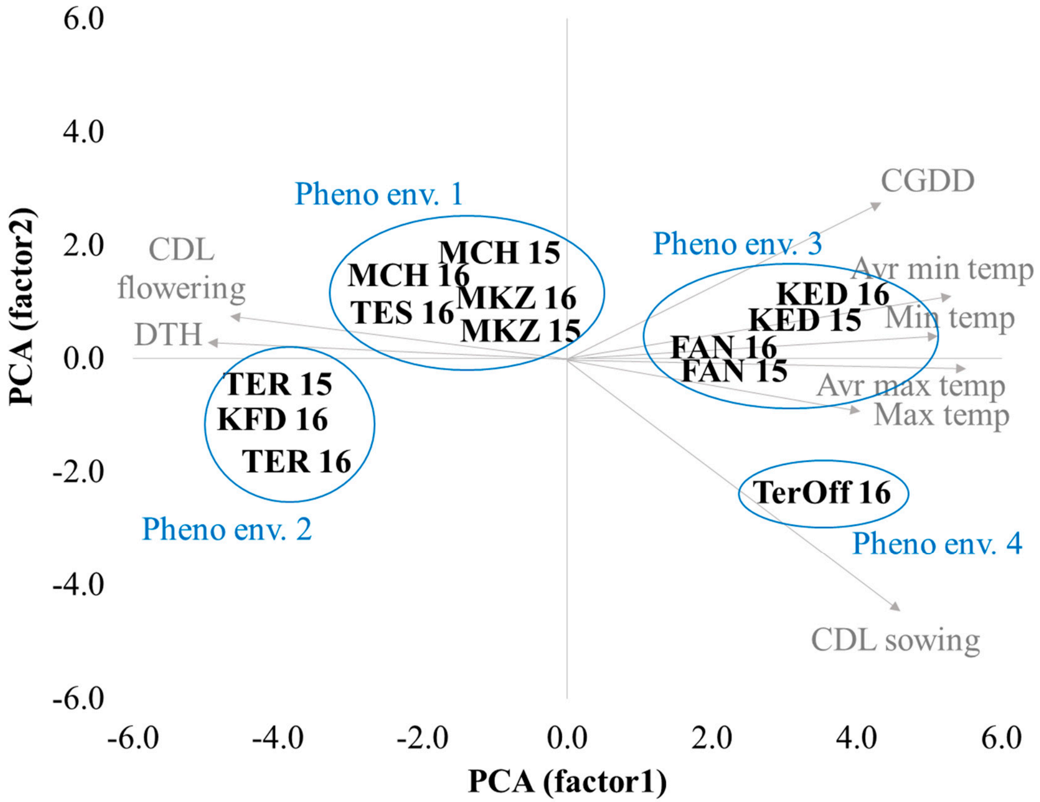

Figure S1: PCA depicting the distribution of four pheno-environments based on climatic and phenotyping data where PCA1 accounts 86.03 % and for PCA2 8.86%

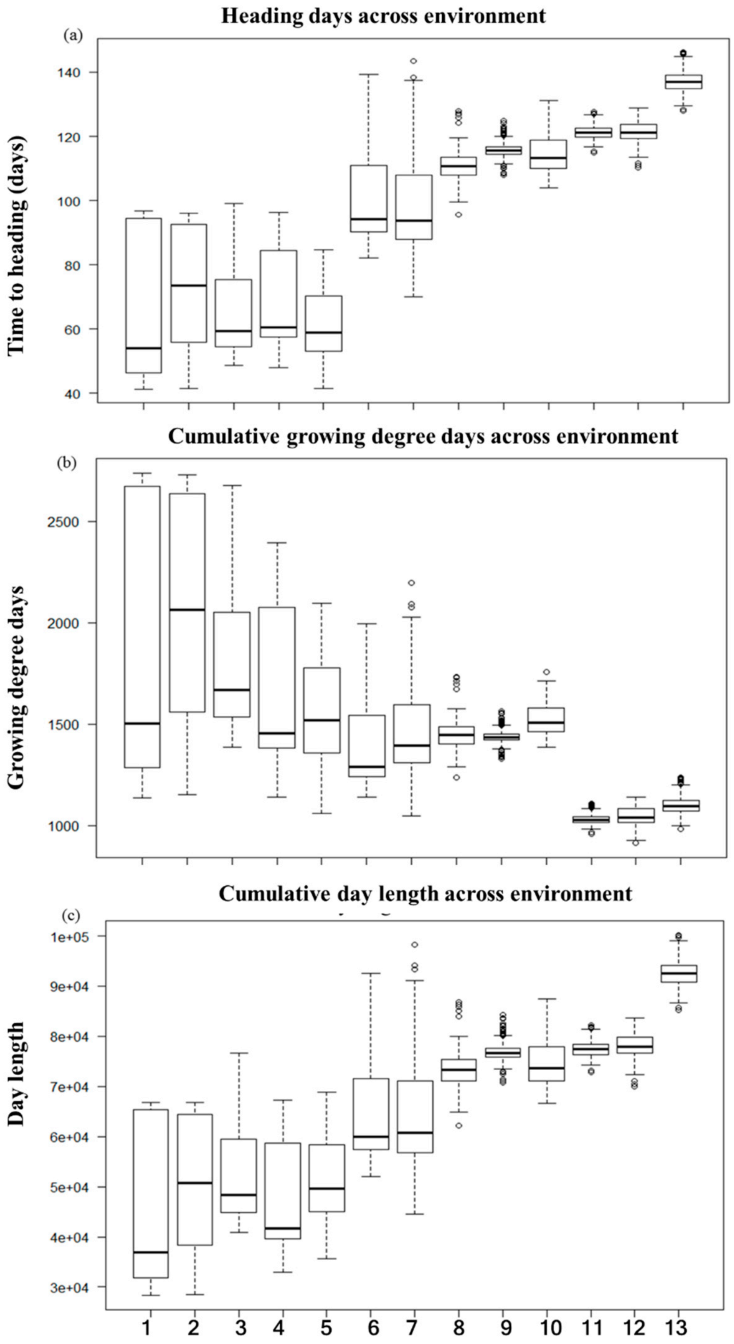

Figure S2: Boxplots showing the distribution pattern of heading days (DTH), Cumulative growing degree days (CGDD), and Cumulative day length (CDL) among 384 durum core collection of ICARDA evaluated at 13 environments namely KED15, KED16, FAN15, FAN16, Teroff, MKZ15, MKZ16, TES16, MCH15, MCH16, KFD16, TER16, and TER15.

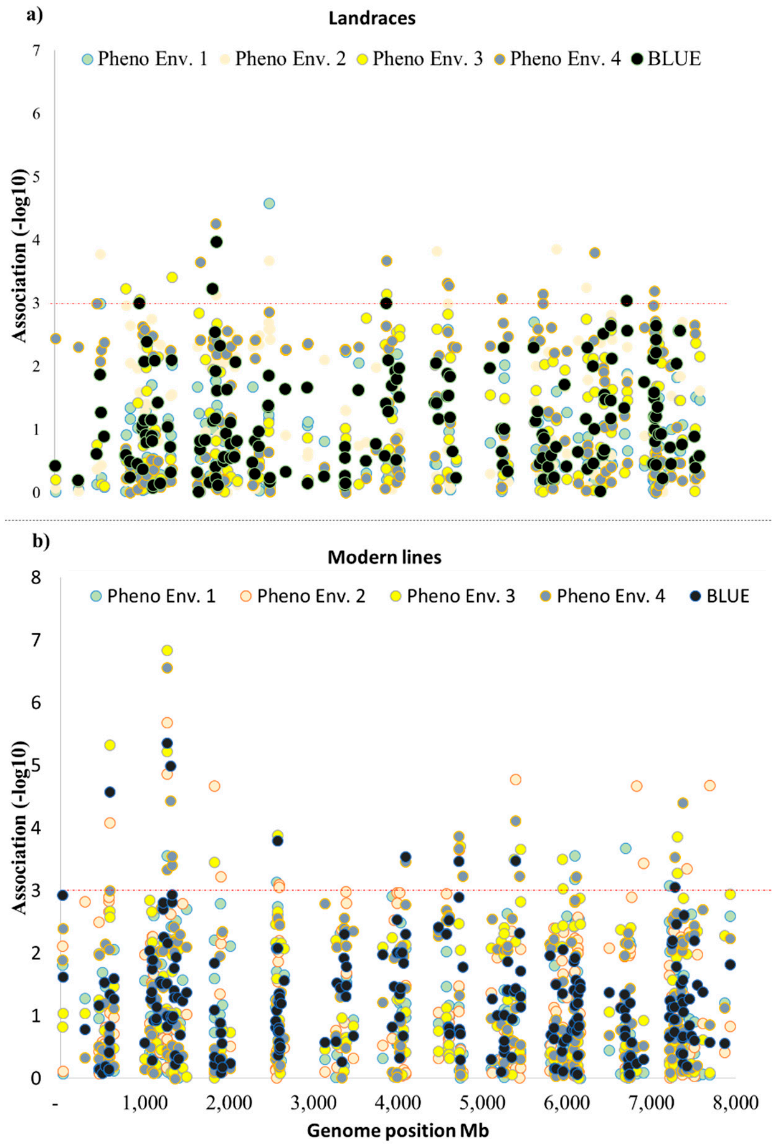

Figure S3: Manhattan plot for marker-trait association for heading days in four pheno-environments and across environments (BLUEs) for (**a**) landraces and (**b**) modern lines of durum wheat. Only loci with LOD superior to zero are presented. Below is the percentage of markers scoring a LOD of 0 for each chromosome.

**0 (%)**	**1A**	**1B**	**2A**	**2B**	**3A**	**3B**	**4A**	**4B**	**5A**	**5B**	**6A**	**6B**	**7A**	**7B**
**Landraces**	54	57	48	48	53	48	48	56	56	52	47	51	46	48
**Modern**	30	34	24	28	37	23	20	25	22	17	21	20	31	26

Table S1: Combined ANOVA (sum of squares) for days to heading, cumulative growing degree days and cumulative day length involving 384 durum lines at 13 environments as well as for four pheno-environments.

Source of variation	df	DTH	CGDD	CDL
Environment	12	4.0E+06**	4.0E+08**	1.3E+12**
Pheno-Environment	3	3.8E+06**	3.8E+08**	1.1E+12**
Error	8	2.0E+05	2.2E+07	1.5E+11
Genotype	383	4.5E+05**	2.2E+08**	2.4E+11**
Genotype X Environment	4,596	4.4E+05**	3.2E+08**	2.2E+11**
Genotype X Pheno-Environment	1,149	3.0E+05**	2.6E+08**	1.5E+11**
Error	936	4.6E+03	1.8E+06	2.4E+09
Proportion of GxE explained by G x PhEnv		68.7%	80.7%	66.6%

**: $p<0.01$, df: degree of freedom, DTH: days to heading, CGDD: cumulative growing degree days, CDL: cumulative day length.

Table S2: Significant QTLs (Bonferroni corrected LOD for *p*<0.05= 3.0, *p*<0.01=3.4) identified among landraces presented with its peak marker ID, chromosome (chr), position, LOD and ratio (%) of phenotypic variance explained.

Locus	Marker	Chr	Position (Mb)	PhEnv 1		PhEnv 2		PhEnv 3		PhEnv 4		Across Env	
				LOD	Var	LOD	Var	LOD	Var	LOD	Var	LOD	Var
Q.ICD.Eps-01	AX-94583506	1A	521.7							3.0	11.8		
Q.ICD.Ppd-02	AX-94635647	1B	317.5					3.2	13.5	3.2	15.5	3.0	17.9
Q.ICD.Eps-03	AX-94963816	2A	556.7							3.7	12.8	3.7	3.6
Q.ICD.Ppd-04	AX-95206454	2A	708.9									3.2	7.0
Q.ICD.Ppd-04	AX-94488406	2A	744.5			3.2	11.8					3.2	2.6
Q.ICD.Ppd-05	AX-94939920	2B	8.6									4.0	8.0
Q.ICD.Ppd-05	Ppd-B1	2B	56.3			3.0	5.7			4.3	8.2		
Q.ICD.Eps-06	AX-94452589	3A	5.4	4.6	10.6	3.7	7.4						
Q.ICD.Eps-07	AX-95021774	3B	765.1					3.2	8.7	3.7	7.2		
Q.ICD.Eps-08	AX-94439386	4A	597.7			3.8	5.4						
Q.ICD.Eps-09	AX-95652066	4A	731.1			3.0	18.1						
Q.ICD.Ppd-10	AX-95101347	4B	12.5							3.3	30.3		
Q.ICD.Vrn-11	Vrn-A1	5A	549.2							3.5	12.0		
Q.ICD.Vrn-12	AX-94939814	5B	392.7			3.9	8.4						
Q.ICD.Eps-13	AX-94930415	6A	195.6									3.1	13.9
Q.ICD.Eps-14	AX-94761286	6A	530.6							3.2	31.1		
Q.ICD.Vrn-15	AX-94451862	6B	686									3.1	3.9
Q.ICD.Vrn-16	AX-94634646	7A	21.1			3.8	3.0						
Q.ICD.Eps-17	AX-95026088	7A	141			3.0	10.4						

Table S3: Significant QTLs (Bonferroni corrected LOD for *p*<0.05= 3.0, *p*<0.01=3.4) identified among modern lines presented with its peak marker ID, chromosome (chr), position, LOD and ratio (%) of phenotypic variance explained.

Locus	Marker	Chr	Position (Mb)	PhEnv 1		PhEnv 2		PhEnv 3		PhEnv 4		Across Env	
				LOD	Var	LOD	Var	LOD	Var	LOD	Var	LOD	Var
Q.ICD.Eps-18	AX-94498055	1B	0.3			4.1	24.1	5.3	6.0	3.0	4.5	4.6	1.6
Q.ICD.Ppd-19	AX-94385320	2A	36.4					6.8	2.6	4.4	6.6	5.0	12.6
Q.ICD.Ppd-19	*Ppd-A1*	2A	36.6	3.6	3.3	5.7	8.1	7.5	1.2	6.6	0.7	8.5	5.5
Q.ICD.Eps-03	AX-94460586	2A	556.8			4.7	0.7	3.5	0.4				
Q.ICD.Ppd-05	AX-94956877	2B	54.2			3.6	3.7					4.2	2.1
Q.ICD.Ppd-05	*Ppd-B1*	2B	56.3			3.6	7.6					4.2	2.0
Q.ICD.Eps-20	AX-94593608	3A	662.4	3.1	2.0								
Q.ICD.Eps-20	AX-94479255	3A	676.8					3.9	1.3			3.8	1.4
Q.ICD.Eps-20	AX-95230073	3A	693.0			3.1	1.9						
Q.ICD.Eps-21	AX-94973426	3B	0.3			3.1	2.0						
Q.ICD.Eps-22	AX-94657503	4A	48.0			3.2	2.0						
Q.ICD.Eps-09	AX-95630216	4A	687.4			3.3	2.1						
Q.ICD.Ppd-10	AX-94554200	4B	26.9							3.5	14.1	3.5	1.2
Q.ICD.Eps-23	AX-94394439	4B	656.5			3.7	7.9			3.9	1.9	3.5	4.3
Q.ICD.Vrn-24	AX-94577903	5A	11.4					3.7	5.0	3.2	4.6		
Q.ICD.Vrn-11	*Vrn1*	5A	549.2					3.0	20.0	3.9	10.0		
Q.ICD.Vrn-25	AX-95213349	5A	644.7					3.5	1.0				
Q.ICD.Vrn-25	AX-94608103	5A	664.3					3.5	3.6	4.1	3.9	3.5	6.4
Q.ICD.Vrn-26	AX-95245953	5B	559.8					3.0	39.8				
Q.ICD.Vrn-26	AX-94531833	5B	700.9	3.6	10.6								
Q.ICD.Eps-27	AX-94707895	6B	6.6	3.7	3.0								
Q.ICD.Eps-28	AX-94805681	6B	136.7			4.7	7.0						
Q.ICD.Eps-29	AX-94414186	6B	220.4			3.4	2.8						
Q.ICD.Vrn-15	AX-94637897	6B	528.6	3.1	2.6								
Q.ICD.Vrn-15	AX-94711490	6B	590.8					3.9	5.1	3.5	1.0	3.1	22.0

Q.ICD.Vrn-16	AX-95080277	7A	3.1							4.4	7.7		
Q.ICD.Vrn-16	*Vrn3*	7A	69.4					3.2	5.2				
Q.ICD.Eps-30	AX-94905964	7A	323.7			4.7	1.8						
Q.ICD.Eps-31	AX-94701740	7B	127.5			4.7	6.8						
Q.ICD.Eps-32	AX-94878591	7B	685.1			3.4	3.0						
